# Assessment of the Reproductive Health Status of Adult Prison Inmates in Osun State, Nigeria

**DOI:** 10.1155/2013/451460

**Published:** 2013-03-26

**Authors:** A. I. Olugbenga-Bello, O. A. Adeoye, K. G. Osagbemi

**Affiliations:** ^1^Department of Community Medicine, Faculty of Clinical Sciences, College of Health Sciences, Ladoke Akintola University of Technology (LAUTECH), P.M.B. 4400, Osogbo, Osun State, Nigeria; ^2^Department of Community Medicine, Ladoke Akintola University of Technology (LAUTECH) Teaching Hospital, P.M.B. 4008, Ogbomoso, Oyo State, Nigeria; ^3^Department of Epidemiology and Community Health, University of Ilorin, Ilorin, Nigeria

## Abstract

*Introduction*. All over the world, numbers of prisoners have being increasing with majority in the sexually active age group; hence diseases such as HIV, Tuberculosis and Hepatitis are more prevalent in prisons than in the community. This study thus aims to provide an overview of the reproductive health status of adult prison inmates in Osun State. *Methodology*. This is a cross-sectional study among adult inmates in Osun State prison. Data was obtained from 209 selected respondents using pre-tested semi structured questionnaire. *Result*. Majority of the respondents were in the age group 20–39 years with mean age of 30.9 + 7.5. 73.2% are aware of STIs, 93.3% HIV/AIDS and 81.3% contraception. 54.6% had multiple sexual partners before incarceration and 23.3% of them used condom always. 89.5% were not involved in any sexual practice inside the prison, 9.1% masturbated and 1.4% had homosexual partners. Less than 6% had access to male condoms gotten from prison staffs and prison clinics. *Conclusion and recommendation*. No comprehensive reproductive health care system to address reproductive health services in prisons. Respondents' knowledge about STIs, HIV/AIDS and contraception is good, but their condom usage is low compared with the knowledge. Government should put in place specific reproductive health programmes in prisons.

## 1. Introduction

In recent times, there has been a marked increase in the number of prisoners in many countries around the world. According to the World Prison Population List, prison populations have increased by 73% over a relatively short period of time [1]. As in any human population, there are several health problems confronting inmates living in prisons all over the world, these include both physical and mental illnesses. Prisons are not closed off worlds; many people (prison staffs, lawyers, social workers, health personnel, the clergy, and prisoners' family members) enter and leave prisons every day. Many prisoners themselves stay only a short period in prison and return to their families. It is estimated, for instance, that although the worldwide prison population is over 9 million, the annual turnover is closer to 30 million [1]. This high movement of people into and out of prisons makes the possibility of infections acquired in prison being transmitted outside very high [2]. In spite of this, gap still exists globally in the provision of health care to prisoners, especially with respect to sexually transmitted infections (STIs), HIV, and other blood borne infections [3–6]. It has been documented that communicable diseases such as HIV, tuberculosis, and hepatitis are more prevalent in prisons than in the community [7, 8]. This creates a crucial public health issue for correctional institutions and at a broader level the communities in which they are situated.

No matter how pleasant wardens and prison staff might be, it might not be easy for an inmate to ask for assistance concerning reproductive health issues; hence the sexual activities and indication for intervention remain hidden [9]. Previous studies show that majority of incarcerated women who are afflicted with STIs and/or HIV are asymptomatic or unwilling to seek medical help [10–12]. Most prisons are meant to offer basic medical care, including reproductive health care, but few correctional facilities have policies that ensure access to such care [13]. Relatively few countries have policies governing access to reproductive health care, and if such policies exist, they varied widely. In most prisons, especially in the developing countries in which Nigeria is included, health care policies are “one size fits all” for both male and female inmates [13].

The Nigerian Prisons Service (NPS) was founded as an institution to correct social deviants, punish and reform criminals and to complement the processes of legal adjudication and law enforcement [14]. The main aim of establishing the prison institution in all parts of the world including Nigeria is to provide rehabilitation and correctional facilities for those who violated the rules and regulations of their society.

However, living conditions in Nigerian prisons are appalling and in many cases constitute clear threats to health. Conditions such as overcrowding, poor sanitation, lack of food and medicines, and denial of contact with families and friends fall short of United Nations standards for the treatment of prisoners. In many Nigerian prisons, inmates sleep two to a bed or on the floor in filthy cells. Toilets are blocked and overflowing or simply does not exist and there might be no running water; diseases are wild spread as a result. Some prisons have small clinics which lack medicine, some have hospitals, but guards often demand that inmates pay bribes for such privileges as visiting the hospital [15]. The Prison system in Nigeria is one of the most under developed institutions in the criminal justice sector [16]. No new prison has been constructed in more than forty years and the prison population continues to grow [17]; in addition to this congestion are poor funding, sexual assaults, corruption, and fraud [16]; all these are cause of decay in the prison system generally, the health sector inclusive.

Nigerian prison has less than 4% of total prison admissions to be females, about 80% serve short-term imprisonment of less than two years, and more than 50% are between the ages of 28 and 50 years [15].

Prisoners' age of between 20 and 40 years old is an age group usually considered to be very sexually active [18, 19]; this, coupled with an excess free time, makes the prison environment conducive for various forms of risky behaviors such as sharing of needles among intravenous drug users, unprotected sex with same sex partners, as well as opposite sex inmates; in fact, sex is often the primary form of entertainment in some prisons [9, 19–21].

In study done in Nepal, it was observed that 71% of prisoners were intravenous drug users and 75% of them always shared needles, 38% had casual partners and 30% had frequent visits to sex workers, none of the inmates ever used condoms, and only 14% perceived that they might have HIV [20]. Also in another study done in India, 76.6% of the male inmates gave history of penetrative sex. Of these 54.2% gave a history of having multiple sexual partners and the majority had unprotected sex, even with commercial sex workers, while 72.5% of them were not aware of HIV [22]. In Nigeria, a study done in Kaduna showed that 14.9% of inmates reported having sexual intercourse in the prison and of these 43.8% claimed to have used condoms regularly [8]; it is thus seen that prisoners engage in sexual activities right inside prisons during incarceration, although one would think this should not be possible because a prisoner's freedom is expected to be limited. 

Consequent to the prevalent risky sexual behaviors observed among prison inmates, various studies have given indications that sexually transmitted and blood-borne infections are highly prevalent in prison premises [9, 22]; these range from hepatitis, active pulmonary tuberculosis (PTB), syphilitic ulcers on the penis and confirmed HIV infection [22], with the prevalence of confirmed HIV cases ranging from 1% to as high as 16.5% [22–25].

The reproductive health of prison inmates is an inevitable part of public Health, because there are interactions between the prisons and the society. HIV has also been found to be consistently higher among prisoners than the general public [20] and the high level of inmate turnover [15] means that HIV transmission in prison threatens HIV control in the community when inmates are released; in addition, incarceration is an opportunity to provide reproductive health services to prisoners who might not otherwise seek health services.

This area of research is important because addressing reproductive health needs of prisoners is essential in any public health initiative that aims to improve overall public health, and since these prisoners engage in various forms of sexual behaviours inside and outside the prison premises [8, 9, 19–21], appropriate reproductive health services for prisons cannot be overemphasized.

The study thus aims to provide an overview of the reproductive health status of adult prison inmates in Osun State, as this will help in formulating proper reproductive health policies and programmes for prisons.

## 2. Methodology

This descriptive cross-sectional study was carried out in Osun State, South Western part of Nigeria. There are two prisons in the state—a medium-sized type in Ilesa, and a smaller capacity type in Ile-Ife town, both in Osun east senatorial district. The study population is adult prison inmates, who were above 18 years of age. The total number of inmates in both prisons in the state during the study period was 464 (337 in Ilesa and 127 in Ile-Ife), and a total of 209 inmates were recruited for the study after calculating the sample size using Leslie-Fischer's formula for the population below 10 000. Respondents were chosen from both prisons using simple random sampling method, and the number of respondents chosen from each prison was based on proportional allocation from the total number of inmates per prison; thus 57 respondents were chosen from Ile-Ife prison, while 152 were chosen from Ilesa. There are no female prisoners in Ile Ife prison, but Ilesa prison has 14 inmates; therefore the respondents at Ilesa prison were stratified based on sex before proportional allocation of the 152 that was chosen; 146 males and 6 females were thus chosen. Approval was sought from the Nigerian prisons services, Osun State, LAUTECH Teaching Hospital, ethical review committee, and the consents of participants were sought after explaining the research details to them, respondents were also told that participation is voluntary, and participation would not in any way influence their incarceration terms. All the respondents approached and recruited for the study participated giving a response rate of one hundred percent.

Pretested semistructured questionnaire was used for data collection, they were self-administered for literate inmates and interviewer's method was used for illiterate respondents by trained research assistants; two offices were designated for carrying out these interviews in order to ensure privacy. Although respondents' privacies were ensured and they were all assured that all information given will not be traceable back to them, possibility for reporting biases cannot be totally ruled out because of the sensitivity of some questions.

The questionnaire obtained information on socio-demographic profile of the inmates, knowledge about HIV/AIDS, other STIs and contraception, sexual risk behaviours of respondents, and access to health care while in prison.

Evaluation of knowledge of respondents about reproductive health care was assessed based on scoring of twenty-one [21] questions that were asked in the questionnaire; a score of one is administered for every right answer while zero is allocated to every wrong answer; the overall mean score obtained was 10 and respondents that scored 10 and above were taken to have good knowledge while respondents that scored below ten were said to have poor knowledge.

Data extracted from questionnaires were analyzed using Statistical Package for Social Sciences (SPSS) Version 15. Descriptive statistics were applied to determine frequency of relevant variables in the study, while inferential statistical method using chi-square was used to test associations between variables such as age, sex, educational status, and period of incarceration, and knowledge about reproductive health care, with *P* value set at <0.05.

## 3. Result

### 3.1. Sociodemographic Characteristics of Respondents

 Out of the 209 respondents recruited for this study, the majority were in the age group 20–29 years ((90) 43.1%), followed by age group 30–39 years, having eighty-three (39.7%) respondents. Just ten inmates (4.8%) are less than 20 years and three (1.4%) are greater than 50 years. The mean age of the respondents is 30.9 ± 7.5.

Males are more predominant; they are two hundred and three (97.1%); majority are also single ((93) 44.5%), have secondary education ((92) 44.0%), and are unemployed before incarceration ((88) 42.1%). One hundred and sixty one (77.0%) of the respondents have their total incarceration period (total length of prison sentence) to be less than or equal to 5 years (Table 1).

### 3.2. Knowledge of Respondents about STIs

One hundred and fifty three (73.2%) of the respondents were aware of STIs. When these 153 were asked about symptoms suggestive of STIs, 76 (49.7%) said painful urination, 26 (17.0%) said genital ulcer, 44 (28.8%) said urethral/vaginal discharge, 61 (39.9%) said genital itching, and 58 (37.9%) said lower abdominal pain. One hundred and thirty (85.0%) of the 153 were also aware of the modes of transmission of STIs among these, they all (100.0%) said transmission could be via blood transfusion and sexual intercourse with opposite sex, 91 (70.0%) said transmission could also be through sexual intercourse with same sex, 59 (45.4%) said it could be through unsterilized needles, and 10 (7.7%) said it could be transmitted by talking to infected person. Forty-nine (23.4%) of our total respondents said they have had one form of STI before (Table 2).

### 3.3. Knowledge about HIV/AIDS

Respondents' knowledge about HIV/AIDS was also assessed; one hundred and ninety five (93.3%) were aware about HIV/AIDS; among these, a hundred and seventy-four (89.3%) said HIV could be transmitted by having unprotected sex with an infected person, 153 (78.5%) said it can be transmitted through injection with unsterilized needles, 110 (56.4%) believed it can be transmitted through use of unsterilized sharps such as clippers and blades 59 (30.3%) said its transmission could be from kissing an infected person, 25 (12.8%) said through mosquito bite, 11 (5.6%) said through sharing of clothes and towel with an infected person, 7 (3.6%) said using same swimming pools/streams, 29 (14.9%) said it could be transmitted spiritually and via witchcraft, 38 (19.5%) believed it could be from homosexuality, and another 7 (3.6%) said transmission could be from eating from same plate with an infected person. 151 (77.4%), 157 (80.5%), 114 (58.5%), and 119 (61.0%) said HIV transmission could be prevented by being faithful to partner, abstaining from sex, not sharing sharps, and barrier contraception, respectively. When asked if respondents have heard about HIV Counseling and Testing (HCT) and know what it means, 84 (40.2%) have heard about this; similarly 84 (40.2%) are aware of anti-retroviral therapy (ART), among which 21 (25.0%) said ARV cures HIV completely, and 2 (2.4%) said ARV does nothing (Table 3).

### 3.4. Knowledge about Contraception and Antenatal Care

 Respondents' knowledge about contraception revealed that 170 (81.3%) of them are the ones that are aware about contraception. Twenty-eight (16.5%) among these respondents who are aware about contraception said that contraception encourages promiscuity, and the same number also believed that it decreases sexual pleasure; 136 (80.0%) said it helps in preventing STIs/HIV; 122 (71.8%) said it is for reducing the number of children to be born, while 106 (62.4%) said it helps in child spacing. All the respondents that are aware of contraception know the male condom ((170) 100.0%) as a form of contraception, 75 (44.1%) know about female condom, 65 (38.2%) each are aware of injectables and IUCD, while only 9 (5.3%) know about implants.

When respondents' knowledge about antenatal care was also assessed, one hundred and thirty-eight (66.0%) of the total respondents are aware of antenatal care, majority of them [(43) 31.2%] got their information about Ante natal care from the radio (Table 4).

Ninety-eight (46.9%) inmates were eventually found to have good knowledge about reproductive health care.

### 3.5. Sources of Information about STIs, HIV/AIDS, and Contraception

Out of the 153 (73.2%) of the respondents that are aware of STIs, equal number ((49) 32.0%) got their information from television and radio each, 21 (13.8%) and 34 (22.2%) have the school and friends/family as their sources respectively, while none got information from the newspaper. Among respondents that were aware about HIV/AIDS (195 (93.3%)), 70 (35.9%), 49 (25.2%), 42 (21.5%), 31 (15.9%), 2 (1.0%), and 1 (0.5%) got their information from radio, television, family/friends, school, newspaper, and hospital, respectively. Respondents' knowledge about contraception also revealed that majority (55 (32.4%)) of the 170 (81.3%) that are aware got their information from the radio, followed by 46 (27.1%) who got theirs from friends/family members (Tables 2, 3 and 4).

### 3.6. Sexual Practices and Reproductive Healthcare of Respondents

Forty-six (22.0%) respondents said they have no sexual partner prior to their incarceration, 49 (23.4%) have one sexual partner, while the remaining 114 (54.6%) have more than one sexual partners ranging from two to more than five. Only thirty-eight (23.3%) among those who have been exposed to sex said they used barrier method of contraception always, while 37 (22.7%) said they have never used. When asked about their sexual practices inside the prison, 19 (9.1%) said they do masturbate, none was involved with the opposite sex partner, while 3 (1.4%) said their sex partners are people of the same sex. One hundred and eighty-seven (89.5%) said they were not involved in any sexual practices inside the prison. Information was also sought about sexual harassment inside the prisons; by this, respondents were asked if another person has attempted to forcefully have sexual intercourse with them. Eight (3.8%) respondents said they have been sexually harassed by opposite sex inmate, 5 (2.4%) have been harassed by same sex inmate, and 1 (0.5%) person has been harassed by the prison staff.

Respondents' use of the prison clinic was sought and only 55 (26.3%) said they have ever been to the prison clinic to take care of their basic health needs; however when inquiry was made about reproductive health care that inmates have access to in the prison, 12 (5.9%) of the respondents were found to have ever had access to male condom; among these, 3 (1.4%) got theirs from prison staffs, while the remaining 9 (4.3%) got from the prison clinic during a health talk about HIV/AIDS by a Nongovernmental Organisations (NGOs); none of them have access to other forms of family planning services. 148 (70.8%) of the respondents have attended seminars/health talks on HIV while in prison, and among these, missioners (40 (27.0%)), prison authorities (35 (23.7%)), and NGOs (33 (22.3%)) were the organizers of these seminars/health talks (Table 5).

After cross-tabulation of sociodemographic characteristics of respondents with their knowledge about reproductive health care, there were statistically significant positive relationships between knowledge and age (*P* = 0.000), educational status (*P* = 0.000), and occupation before incarceration (*P* = 0.000); that is, there were more respondents with good knowledge among higher age group, respondents with higher educational status, and professionals and skilled workers. There was also statistically significant relationships between knowledge and marital status (*P* = 0.000), with more married and divorced having better knowledge than singles (Table 6).

## 4. Discussion

About eighty percent of our respondents were in the sexually active age group (20–39 years); this has also been seen in previous studies [18, 19]. This portrays the characteristic age range of prisoners in Nigeria generally [15], and in Osun State as well; the respondents were good representation of the study population (prison inmates in Osun State). Most prisoners being in this sexually active age group show the importance of having reproductive health policies for our prisons.

Over ninety percent of our respondents were also found to be males; this is not however surprising because the female prisoners in the state are very few compared to the males. Although female prisoners have been increasing lately, especially in the United States [12, 26], most prisons still have more males than females [13, 18], as it is also seen in this study. This has made most policies on provision of health care for inmates to be uniform, regardless of gender, ignoring the fact that women require specific health care services, such as routine gynecological examinations or pregnancy screening [13], prison is seen basically as a “man's world”, and despite the fact that females have more heath issues, as well as more difficulties accessing health services during incarceration than male prisoners, prisons' medical systems are mainly designed for men [27, 28]. Females find themselves in a system essentially run by men for men; hence their specific health care and hygiene needs are not taken into account [28]. Attention should therefore be paid to female health care, especially their reproductive health care in prisons, in spite of their small population. Government and policy makers should not be carried away by the males' dominance in prisons and forget to put appropriate health care needs of females in place.

Almost half of our respondents have secondary school education, showing that they have an appreciable level of formal education; the implication of this is that educative programmes about reproductive health services for prisoners should be channeled for both illiterates and literate; previous survey showed that no literacy institute has been established for inmates in the NPS system [16]; although there has been various ongoing reform programmes [29], introduction of education and/or skill acquisition programme is not part of the programme [16]. It is believed that such education programmes, beyond rehabilitating prisoners, will also impact on their health positively.

Almost eighty percent of our respondents are spending five years or lesser in the prison, indicating that most of these inmates would be out of incarceration soon to join the outer society. The high turnover rate of prisoners [1] has been said to make the possibility of infections (STIs inclusive) acquired in prisons being transmitted outside very high [2], thus posing a risk to the control of these diseases.

Approximately three quarter of the inmates interviewed were aware of STIs, and none of them got their information from the newspaper. Maybe these respondents do not read newspapers or the print media hardly discuss such issues as STIs. Over eighty percent of those aware about STIs know the various modes of transmission such as blood transfusion, sexual intercourse with opposite sex, and same sex and sharing of unsterilized needles, but knowledge gap was still found among less than eight percent of them that said transmission could be from talking with infected persons. The majority of the respondents were also aware of symptoms suggestive of STIs (painful micturition, genital itching, and lower abdominal pain); they were however not asked whether STIs could be asymptomatic or not to further assess the extent of their knowledge about STIs as some of these STIs have been found to be asymptomatic; this is a limitation that further studies could explore.

Almost twenty-five percent of the total respondents in this study said they have had STI before; the Nigeria Demography and Health Survey (NDHS) showed just two percent of females and one percent of males to have reported having an STI [30]; the higher prevalence in this study is not surprising as prisoners have been shown to engage in various forms of unprotected sexual behaviours inside and outside the prison premises [8, 9, 19–21], which expose them to these STIs.

Most of the respondents are aware about HIV/AIDS, with the radio being their main source of information. Many of them also know the possible routes of transmission of the virus, such as unprotected sex with infected person, injection with unsterilized needles, and use of unsterilized sharps, and so forth; some however still erroneously believe that transmission could be through mosquito bite, sharing of clothes and towels, usage of same swimming pools with an infected person, witchcraft, and eating from same plate with an infected person. Less than half of the respondents are aware of antiretroviral therapy, and a quarter of these people said the ART is used to totally cure HIV infection. All these responses show that the information some of the inmates have about HIV/AIDS was incorrect.

In addition, respondents were also observed to have a number of misconceptions and myths about contraception; about a third of those that are aware of contraception believed that contraception encourages promiscuity and decreases sexual pleasure. This knowledge gap about contraception poses a great risk for the control of STIs including HIV/AIDS, because condom promotion has been seen as one of the standard STI control interventions. There is strong evidence that male latex condoms reduce transmission of HIV by at least 80%–85%; they are effective against other STIs and even reduce the risk of unintended pregnancies [31]. Other barrier methods of contraception such as female condom also help in prevention of STIs [32].

More than half of the respondents had more than one sexual partners before they were incarcerated, and only about twenty percent of them said they always use barrier method of contraception; this is surprising because despite having many of the respondents to be aware of STIs and its transmission routes, over fifty percent of them are still engaged in high-risk sexual behaviours (multiple sexual partners and nonusage of barrier contraception) that is capable of transmitting these STIs; similar observation was seen among prisoners in Nepal and India where inmates have unprotected sex with multiple sexual partners including commercial sex workers [20, 22], but the Kaduna study differs as almost half of the respondents in this study who were having sexual intercourse in the prison used condom regularly [8]. The nonusage of the barrier contraception in our study might be due to the myth and misconceptions the respondents have had about contraception that it reduces sexual pleasure. It is therefore very important for proper reorientation and education about the importance of contraception. Beyond the misconceptions about contraceptive use, there may also be some other factors that are responsible for the high-risk sexual behaviour among the respondents in spite of their awareness about STIs, more researches may be necessary to explore these other factors. 

While in the prison none of the respondents had sexual intercourse with opposite sexual partner, one percent had sex with same sex partner. Sexual exposure to opposite sexual partner may be difficult because the cells are gender specific, thus preventing such clandestine relationships between opposite sexes; however, the same sex practices may be possible since inmates of the same sex sleep together in the same cell. Contrary to our study however, previous studies reported more sexual activities in prisons [8, 9, 19]; this may be possible as there have been reported cases of guards being bribed for inmates to have such privileges [15]. Presently, the Nigerian law forbids homosexuality, with imprisonment as punishment [33]; although no one has been convicted since the passage of this law, inmates involved in this act are at the risk of serving another jail term if caught and convicted. In addition to this is the risk of higher rate of HIV transmission in homosexuals compared with heterosexuals [34, 35]; hence inmates involved in homosexuality, though small, are more capable of transmitting HIV infection than those who are not.

Few of the prison inmates too have experienced one form of sexual harassment or the other, with one of them being sexually harassed even by a prison staff. Prisoners, especially female ones, have been said to become vulnerable and open to sexual assaults because of promises of favour and special treatments, usually from staffs and even inmates [16].

Another interesting finding in this study is that less than one-third of the respondents have ever used the clinic to care for their basic health needs and none have access to other forms of family planning services except for less than a tenth that have access to male condoms, which they got from either the prison officers or prison clinics during a health campaign on HIV/AIDS by on NGO. Although the prisons used in this study have clinics that take care of basic health needs of prisoners such as malaria and diarrhoeal diseases, special health care needs such as reproductive health needs are not in place in these clinics, and these clinics even lack drugs, a norm in NPS clinics [15]; this explains why many of the respondents do not go to the clinics.

## 5. Conclusion and Recommendation

The study shows that there is no comprehensive reproductive health care system which addresses various reproductive health needs in our prisons.

Knowledge of respondents about STIs, HIV/AIDS, and contraception is quite good, but some still have misconceptions about condom usage as a barrier method of contraception which was reflected in their usages of condom that is found not to be at par with their knowledge.

Government should therefore put in place specific reproductive health programmes in prisons; this is very important as the majority of this group of people are in the sexually active age group. Efforts should be made to educate prisoners more about HIV/AIDS and other STIs so that they will have the right or correct information; the importance of barrier contraception use in the prevention of these STIs (including HIV/AIDS) should also be continually emphasized.

## Figures and Tables

**Table 1:** Sociodemographic characteristics of respondents.

Sociodemographic characteristics	Frequency (*n* = 209)	Percentage (%)
Age groups (years)		
<20	10	4.8
20–29	90	43.1
30–39	83	39.7
40–49	23	11.0
≥50	3	1.4
Sex		
Male	203	97.1
Female	6	2.9
Marital status		
Single	93	44.5
Married	46	22.0
Divorced	28	13.4
Separated	42	20.1
Educational status		
No formal education	28	13.4
Primary	61	29.2
Secondary	92	44.0
Tertiary	28	13.4
Occupation before incarceration		
Unemployed	88	42.1
Unskilled	57	27.3
Skilled	46	22.0
Professional	18	8.6
Tribe		
Yoruba	175	83.7
Igbo	24	11.5
Hausa	6	2.9
Others	4	1.9
Religion		
Islam	98	46.9
Christianity	99	47.4
Traditional	12	5.7
Family type		
Monogamous	130	62.2
Polygamous	79	37.8
Total duration of incarceration (years)		
≤5	161	77.0
6–10	38	18.2
11–15	9	4.3
>15	1	0.5

**Table 2:** Knowledge about STIs.

Variable	Frequency (*n*)	Percentage (%)
Awareness about STIs (*n* = 209)		
Yes	153	73.2
No	56	26.8
Source of Information (*n* = 153)		
School	21	13.8
Television	49	32.0
Radio	49	32.0
Newspaper	0	0
Friends/family	34	22.2
Awareness about transmission modes (*n* = 153)		
Yes	130	85.0
No	23	15.0
Transmission modes known (*n* = 130; multiple respondents)		
Blood transfusion	130	100.0
Same sex intercourse	91	70.0
Talking with infected person	10	7.7
Sharing unsterilized needles	59	45.4
Symptoms suggestive of STI (*n* = 153; multiple respondents)		
Painful urination	76	49.7
Genital ulcer	26	17.0
Urethral/vaginal discharge	44	28.8
Genital itching	61	39.9
Lower abdominal pain	58	37.9
Have you ever had an STI before (*n* = 209)		
Yes	49	23.4
No	160	76.6

**Table 3:** Knowledge about HIV/AIDS.

Variable	Frequency (*n*)	Percentage (%)
Awareness about HIV (*n* = 209)		
Yes	195	93.3
No	14	6.7
HIV information source (*n* = 195)		
School	31	15.9
Television	49	25.2
Radio	70	35.9
Newspaper	2	1.0
Friends/family	42	21.5
Hospital	1	0.5
HIV transmission modes (*n* = 195; multiple respondents)		
Unprotected sex with infected person	174	89.3
Injection with unsterilized needles	153	78.5
Use of unsterilized sharps (clippers, blades, etc.)	110	56.4
Kissing infected person	59	30.3
Mosquito bite	25	12.8
Cloths and towel sharing	11	5.6
Using same swimming pools/streams	7	3.6
Spiritually/through witchcraft	29	14.9
Homosexuality	38	19.5
Eating from same plate	7	3.6
How can HIV be prevented (*n* = 195; multiple respondents)		
Being faithful to partner	151	77.4
Abstaining from sex	157	80.5
Not sharing sharps	114	58.5
Barrier contraception	119	61.0
Awareness about HCT (*n* = 209)		
Yes	84	40.2
No	125	59.8
HCT information source (*n* = 84)		
School	18	21.4
Television	18	21.4
Radio	16	19.1
Friends/family	19	22.6
Hospital	13	15.5
Awareness about ART (*n* = 209)		
Yes	84	40.2
No	125	59.8
What do you think ART does? (*n* = 84)		
Cure HIV completely	21	25.0
Slows disease progression	61	72.6
It does nothing	2	2.4
I do not know	0	0

**Table 4:** Knowledge of respondents about contraception and Ante-natal care.

Variable	Frequency (*n*)	Percentage (%)
*Contraception *		
Awareness (*n* = 209)		
Yes	170	81.3
No	39	18.7
Information sources (*n* = 170)		
School	20	11.8
Television	43	25.3
Radio	55	32.4
Newspaper	1	0.6
Friends/family	46	27.1
Churches/mosques	1	0.6
Hospital	4	2.4
Perception (*n* = 170; multiple respondents)		
Encourages promiscuity	28	16.5
Prevents STIs/HIV	136	80.0
Helps in reducing number of children	122	71.8
Helps in child spacing	106	62.4
Decreases sexual pleasure	28	16.5
Contraception types known (*n* = 170; multiple respondents)		
Male condom	170	100
Female condom	75	44.1
Diaphragm	22	12.9
Injectables	65	38.2
OCP	85	50
IUCD	65	38.2
Implants	9	5.3

*Antenatal care *		
Awareness (*n* = 209)		
Yes	138	66.0
No	71	34.0
Information sources (*n* = 138)		
School	17	12.3
Television	26	18.8
Radio	43	31.2
Friends/family	36	26.1
Churches/mosques	2	1.5
Hospital	14	10.1
Services offered in ANC (*n* = 138; multiple respondents)		
Health education	90	65.2
PCV	93	67.4
Urinalysis	80	58.0
Blood pressure monitoring	79	57.3
HCT	34	24.6
Fundal height monitoring	33	23.9
Immunization	74	53.6
Family planning education	19	13.8

**Table 5:** Sexual practices and reproductive health care of respondents.

Variable	Frequency (*n*)	Percentage (%)
*Sexual practices *		
Number of sexual partners before incarceration (*n* = 209)		
None	46	22.0
1	49	23.4
2	62	29.7
3	28	13.4
4	16	7.7
5	5	2.4
>5	3	1.4
How often barrier method is used (*n* = 163)		
Always	38	23.3
Occasionally	55	33.7
Rarely	33	20.3
Never	37	22.7
Sexual practices inside prison (*n* = 209)		
Masturbation	19	9.1
Sex with opposite sex partner	0	0.0
Sex with same sex partner	3	1.4
None	187	89.5
Sexual harassment experience inside prison (*n* = 209)		
No	195	93.3
By opposite sex inmate	8	3.8
By same sex inmate	5	2.4
By others (wardens/prison staffs)	1	0.5

*Reproductive health care in the prison *		
Access to male condom and source (*n* = 209)		
No	197	94.3
Yes, from prison officers	3	1.4
Yes, from prison clinics (during HIV campaign programme)	9	4.3
Access to other family planning services (*n* = 209)		
Yes	0	0.0
No	209	100.0
Access to seminar/health talk on HIV in prison (*n* = 209)		
Yes	148	70.8
No	61	29.2
Organizers of health talk (*n* = 148)		
Government	29	19.6
Prison authority	35	23.7
Missioners	40	27.0
NGO	33	22.3
Do not know	11	7.4
Use of clinic that takes care of health needs (*n* = 209)		
Yes	55	26.3
No	154	73.7

**Table 6:** Relating sociodemographic characteristics of respondents with knowledge about reproductive health care.

Socio-demographic characteristics	Good knowledge	Poor knowledge	df	*P* value
Age groups (years)				
<20	1 (10.0)	9 (90.0)	4	0.000
20–29	31 (34.4)	59 (65.6)		
30–39	46 (55.4)	37 (44.6)		
40–49	18 (78.3)	5 (21.7)		
≥50	2 (66.7)	1 (33.3)		
Sex				
Male	93 (45.8)	110 (54.2)	1	0.017
Female	5 (83.3)	1 (16.7)		
Marital status				
Single	28 (30.4)	65 (69.6)	3	0.000
Married	28 (60.9)	18 (39.1)		
Divorced	17 (60.0)	11 (40.0)		
Separated	25 (59.5)	17 (40.5)		
Educational status				
No formal education	2 (7.1)	26 (92.9)	3	0.000
Primary	11 (17.3)	50 (82.7)		
Secondary	57 (62.0)	35 (38.0)		
Tertiary	27 (96.4)	1 (3.6)		
Occupation before incarceration				
Unemployed	25 (28.4)	63 (71.6)	3	0.000
Unskilled	23 (40.4)	34 (59.6)		
Skilled	33 (71.7)	13 (28.3)		
Professional	17 (94.4)	1 (5.6)		
Family type				
Monogamous	58 (44.6)	72 (55.4)	1	0.007
Polygamous	53 (67.3) 79	26 (32.7)		
Duration of incarceration (years)				
≤5	68 (42.2)	93 (57.8)	3	0.007
6–10	25 (65.8)	13 (34.2)		
11–15	5 (55.6)	4 (44.4)		
>15	0 (0.0)	1 (100.0)		
